# Analytical validation of quantitative SARS-CoV-2 subgenomic and viral load laboratory developed tests conducted on the Panther Fusion® (Hologic) with preliminary application to clinical samples

**DOI:** 10.1371/journal.pone.0287576

**Published:** 2023-06-29

**Authors:** Ines Lakhal-Naouar, Holly R. Hack, Edgar Moradel, Amie Jarra, Hannah L. Grove, Rani M. Ismael, Steven Padilla, Dante Coleman, Jason Ouellette, Janice Darden, Casey Storme, Kristina K. Peachman, Tara L. Hall, Mark E. Huhtanen, Paul T. Scott, Shilpa Hakre, Linda L. Jagodzinski, Sheila A. Peel

**Affiliations:** 1 Diagnostics and Countermeasures Branch, Walter Reed Army Institute of Research, Silver Spring, Maryland, United States of America; 2 Henry M. Jackson Foundation for the Advancement of Military Medicine, Bethesda, Maryland, United States of America; 3 Moncrief Army Health Clinic, Fort Jackson, South Carolina, United States of America; 4 Emerging Infectious Diseases Branch, Walter Reed Army Institute of Research, Silver Spring, Maryland, United States of America; Children’s National Hospital, George Washington University, UNITED STATES

## Abstract

**Objective:**

Validate the performance characteristics of two analyte specific, laboratory developed tests (LDTs) for the quantification of SARS-CoV-2 subgenomic RNA (sgRNA) and viral load on the Hologic Panther Fusion^®^ using the Open Access functionality.

**Methods:**

Custom-designed primers/probe sets targeting the SARS-CoV-2 Envelope gene (E) and subgenomic E were optimized. A 20-day performance validation following laboratory developed test requirements was conducted to assess assay precision, accuracy, analytical sensitivity/specificity, lower limit of detection and reportable range.

**Results:**

Quantitative SARS-CoV-2 sgRNA (LDT-Quant sgRNA) assay, which measures intermediates of replication, and viral load (LDT-Quant VLCoV) assay demonstrated acceptable performance. Both assays were linear with an R^2^ and slope equal to 0.99 and 1.00, respectively. Assay precision was evaluated between 4–6 Log10 with a maximum CV of 2.6% and 2.5% for LDT-Quant sgRNA and LDT-Quant VLCoV respectively. Using negative or positive SARS-CoV-2 human nasopharyngeal swab samples, both assays were accurate (kappa coefficient of 1.00 and 0.92). Common respiratory flora and other viral pathogens were not detected and did not interfere with the detection or quantification by either assay. Based on 95% detection, the assay LLODs were 729 and 1206 Copies/mL for the sgRNA and VL load LDTs, respectively.

**Conclusion:**

The LDT-Quant sgRNA and LDT-Quant VLCoV demonstrated good analytical performance. These assays could be further investigated as alternative monitoring assays for viral replication; and thus, medical management in clinical settings which could inform isolation/quarantine requirements.

## Introduction

Coronavirus Disease 2019 (COVID-19) is an ongoing global pandemic caused by infection with the severe acute respiratory syndrome coronavirus 2 (SARS-CoV-2). Fast genome mapping of SARS-CoV-2 has accelerated the development of multiple real-time reverse transcription polymerase chain reaction (RT-PCR) assays that have become the gold standard for detection of viral ribonucleic acid (RNA) and the identification of patients with COVID-19 as well as asymptomatic carriers [1]. Asymptomatic infection accounts for about 35% (95% confidence Interval: 30.7 to 39.9%) of COVID-19 cases [2]. The pandemic has highlighted the importance of rapid and reliable/diagnostic testing to detect infected individuals and limit pandemic spread [3] with high throughput testing continuing to play a major role. Individuals frequently test positive for viral fragments beyond 6 weeks of onset of symptoms, in a fluctuating positive/negative pattern [4]. Moreover, a positive result as detected by quantitative real-time PCR (RT-qPCR) for viral genomic sequences does not attest to the presence of replication-competent virus, since viral fragments may remain after viral clearance [5–7] and thus, does not identify infectious potential. There is a compelling need for tests capable of characterizing infectivity, not just RNA detection.

A key component of the prevention of SARS-CoV-2 transmission is the early identification and isolation of infected individuals with the highest probability of transmitting the virus (most infectious). Determination of infectiousness potential relies mainly upon viral culture as the gold standard, but this capability is not suitable, nor amenable to high throughput. Moreover, it carries an evident infection risk to staff; furthermore, few diagnostics laboratories contain Biosafety Level 3 (BSL-3) facilities to undertake such an effort [8]. Assessment of subgenomic RNA (sgRNA) has been described as a molecular tool to measure infectious potential [9]. sgRNAs are produced by the discontinuous transcription of virion structural genes during active replication and result in the formation of rearranged template sequences that are not found in juxtaposition in the native RNA genome of the virus [10]. Several canonical sgRNAs were identified in SARS-CoV-2-infected Vero cells [11] as part of a high-resolution map of the SARS-CoV-2 transcriptome, epitranscriptome, and in the annotated genome as well [12]. sgRNAs encode the conserved structural proteins spike (S), envelope (E), membrane (M), nucleocapsid (N) and accessory proteins open reading frame (ORF) 3a, 6, 7a, 7b, 8, and 10. Since sgRNA is transcribed only in infected cells and is not packaged into virions, sgRNA levels reflect virus replication [13]. Recently, sgRNA was described as a useful tool for assessing viral infectivity for organ transplant [14] and monitoring the virological response in patients who receive remdesivir [15].

The potential utility of sgRNA to identify intermediates of replication (potentially indicative of actively replicating virus) prompted development of high throughput automated specimen-to-answer quantitative sgRNA and Viral Load (VL) assays (based on the E target) on the Hologic Panther Fusion^®^ platform using its Open Access Functionality. Several Laboratory Developed Tests (LDTs) targeting different pathogens including SARS-CoV-2 [16] were developed on the Panther Fusion^®^ to allow for simultaneous detection of norovirus and rotavirus [17], subtyping of Influenza A Virus (FluA) [18], characterization of respiratory tract infection with Influenza A virus (Flu) A, FluB, and Respiratory Syncytial Virus (RSV) [19], and detection/differentiation of pandemic and endemic coronaviruses (Human Coronaviruses (HCoV) NL63, 229E, HKU1, OC43 and SARS-CoV-2) [20]. Panther Fusion^®^ SARS-CoV-2 LDTs targets included ORF1a (Hologic Food and Drug Administration Emergency Use Authorization), the RNA-dependent polymerase gene [21] and E + N1 [22]. However, our novel LDT is the first quantitative assay and the first to use sgRNA as the target.

In this study, the analytical performance of two LDTs, the SARS-CoV-2 LDT-Quant sgRNA and LDT-Quant VLCoV assays was evaluated.

## Material and methods

### Laboratory developed test protocol

Testing was conducted on the Panther Fusion® random access platform. myAccess software (Marlborough, MA, USA; Panther Fusion^®^ Open Access™ feature) was used to define the PCR protocol, RNA thermal profile (S1 Table), specify target names, choose extraction reagent and detection channels, and set standard curve parameters. Two Laboratory Developed Test (LDT) protocols were created and termed LDT-Quant sgRNA (sgRNA) and LDT-Quant VLCoV (Viral Load) assays. Panther Fusion^®^ Extraction Reagent-S, used for total nucleic acid extraction, contains an internal RNA control (IC) which is used to monitor the extraction process, reverse transcription, and PCR (IC ASR for Internal Control Analyte Specific Reagent, Table 1). Sample input for extraction was 360 μL (derived from 500 μL Nasopharyngeal swab in 710 μL Specimen Transport Media: STM, Hologic ASY-10778, Marlborough, MA, USA). Sample aspiration height was set to “medium” and total elution volume was 50 μL. The template volume for each Polymerase Chain Reaction (PCR) was 5 μL. The universal RNA/DNA cartridge was used. sgE and VL targets (E gene, Table 1) for the LDT-Quant sgRNA (using sgLead SARSCoV2-F, TAL E3 gene R and TAL E3 P) and LDT-Quant VLCoV (using TAL E3 gene F, TAL E3 gene R and TAL E3 P) assays were analyzed in the FAM (6-carboxy-fluorescein) channel and IC in the Quasar 705 channel. Fluorescence was detected during the annealing/extension step. A minimum of one positive channel (IC) was required for a valid result.

**Table 1:** Sequences for the LDT-Quant VLCoV and LDT-Quant sgRNA assays.

Product	Name	Source	Sequence	Position	5’ Modification	3’ Modification
**Forward Primer**	TAL E3 gene F	This study	CGTGGTATTCTTGCTAGTT	26,313 to 26,331	None	None
**Forward Primer**	sgLead SARSCoV2-F	Wolfel, 2020 [9]	CGATCTCTTGTAGATCTGTTCTC	44 to 66	None	None
**Reverse Primer**	TAL E3 gene R	This study	AACGTAAAAAGAAGGTTTTACAAG	26,395 to 26,418	None	None
**Probe**	TAL E3-P	This study	CTAGCCATCCTTACTGCGCTTCG	26,335 to 26,357	FAM	BHQ-1 (Black Hole Quencher 1)
**IC Primers Forward/Reverse**	RNA IC Primers (ASR)	Hologic	Proprietary to Hologic (Cat # PRD-04307)	NA	None	None
**IC Probe**	RNA IC Probe (ASR)	Hologic	Proprietary to Hologic (Cat # PRD-04309)	NA	Quasar 705	Proprietary

NA: Not Applicable

### Primers Probes Reagents (PPR)

Primers and probes (designed by Allele ID version 7.81; Premier BioSoft), for the LDT-Quant sgRNA and LDT-Quant VLCoV assays targeted the SARS-CoV-2 envelope gene (Table 1). Primers and probes (LGC Biosearch Technologies, Middlesex, UK) and salt concentrations were optimized for each primer/probe reagent (PPR) and are summarized in S2 Table. Salts and IC ASR primers and probes were provided by Hologic.

### Calibrators preparation

Calibrators for the LDT-Quant sgRNA and LDT-Quant VLCoV assays were contrived using 7 concentrations (1E9 to 1E3 copies/mL; E is for exponential) of the armored RNA Quant SARS-CoV-2 panel (Asuragen, Cat# 52036, Austin, TX, USA) in STM (Hologic ASY-10778, Marlborough, MA, USA). Calibrators were run in triplicate; standard curves were generated on myAccess software, then imported to the Panther-Fusion LDT protocols.

### LDTs assay controls

Negative Controls for both LDTs were prepared using STM. High and Low extraction controls for the LDT-Quant VLCoV were contrived using 1E6 and 1E4 copies/mL of the heat inactivated SARS-CoV-2 virus (HI-SARS-CoV-2: VR-1986HK™, ATCC, Manassas, VA, USA, respectively). High and Low extraction controls for the LDT-Quant sgRNA were prepared using the Quantified armored RNA consisting of the sgRNA E gene target sequence (ARQ sgE) custom made for the study by Asuragen (Austin, TX, USA).

### Test materials

Precision, linearity, and lower limit of Detection (LLOD) panels for the LDT-Quant sgRNA LDT-Quant VLCoV and assays were prepared using dilutions of HI-SARS-CoV-2 or ARQ sgE, respectively. The specificity panel contained commerical common respiratory flora and other viral pathogens (Zeptometrix, Buffalo, NY, USA or ATCC, Manassas, VA, USA) (S3 Table) added to STM and Viral Transport Media (VTM) diluent (replicating the ratio in a patient sample submitted to the Panther Fusion^®^: 500 μL specimen in 710 μL STM or VTM). A second set of interference samples was also contrived with the addition of 1E4 Copies/mL HI SARS-CoV-2 (HI-SARS-CoV-2: VR-1986HK™, ATCC, Manassas, VA, USA) to create a sensitivity interference panel.

For validation of test accuracy, previously tested specimens covering different cycling threshold (Ct) ranges (as determined by the Hologic Panther Fusion^®^ EUA assay) were obtained from Walter Reed Army Institute of Research (WRAIR, MD, USA) #2790, an enhanced surveillance public health project. Samples from participants who gave permission for future research use of their samples (collected from October 14 to November 23, 2020) were then tested as de-identified samples under WRAIR Protocol #2769.02 (July 2021). The studies involving human participants were reviewed and approved by Institutional Review Board at WRAIR. In total, 70 positive and 100 negative nasopharyngeal swab specimens from U.S. Army trainees isolated for COVID-19 were included [23, 24].

### Data analysis

Data analysis was performed using EP Evaluator (version 12.1.0.18, Data Innovations, Colchester, VT, USA), Prism GraphPad (Version 9.3.0, San Diego, CA, USA) or Python Version 3 (Fredericksburg, VA, USA). Linearity was assessed in CLSI EP6 Linearity module based on Log_10_ Copies/mL. Probit analysis for the LDTs (LLOD determination) was performed in Python using a script developed in-house. The LLOD was set at the concentration where ≥ 95% of the samples are positive.

## Results

### Assays calibration

**Fig 1:**
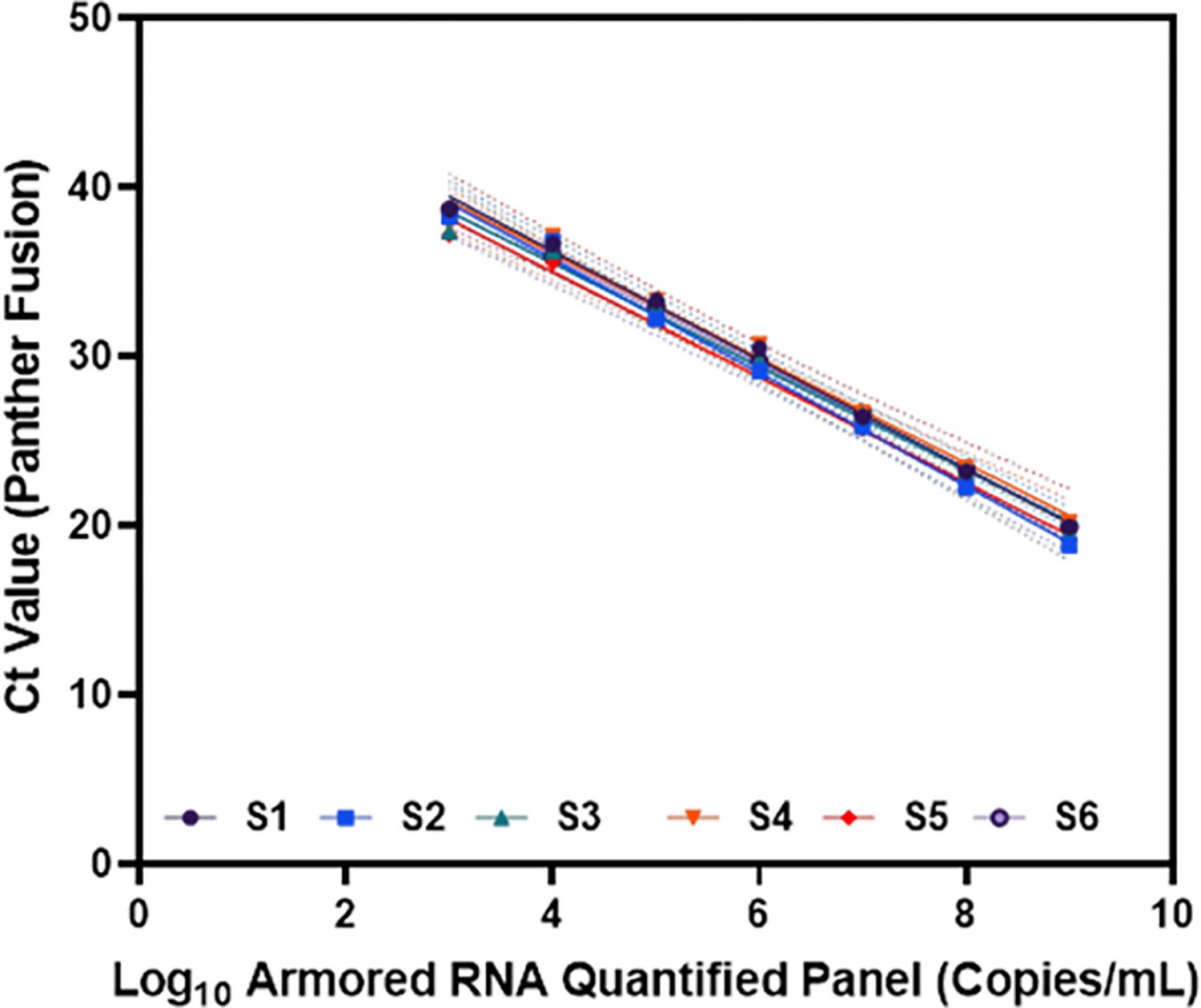
Summary of calibration runs. For every calibration run, a standard curve (shown as S1 to S6, S is for standard curve) for the Ct values obtained for 7 serial dilutions of armored RNA quantified panel (1E9 to 1E3 Copies/mL) was plotted in Prism.

A series of calibrators ranging from 1E9 to 1E3 SARS-CoV-2 RNA input copies (Armored RNA quantified panel) was used to generate the standard curves for the LDT-Quant VLCoV and LDT-Quant sgRNA Panther Fusion^®^ protocols. The assays demonstrated a broad linear, dynamic range from 1E3 to 1E9 input copies with an R squared (R^2^) = 0.99 (Fig 1, S4 Table). The calibrator Ct values demonstrated good precision over 6 runs ranging from 1.5 to 2.7% CV (S4 Table). Calibration acceptability was -3.16 (± 0.33) for the slope and 48.4 (±2.07) for the Y- intercept (S4 Table).

### Novel LDT-Quant VLCoV and LDT-Quant sgRNA assays specificity / interference

To assess the specificity of the LDT-Quant VLCoV and LDT-Quant sgRNA assays, a panel of 29 organisms including common respiratory flora and other viral pathogens was selected. All assessed organisms were Not Detected (TND: Target Not Detected) by the LDT-Quant VLCoV or LDT-Quant sgRNA assays; internal control (IC) values did not demonstrate the presence of any inhibition (Table 2). Moreover, when assessed for interference, no pathogen interfered with the detection or quantification of viral load (LDT-Quant VLCoV) or IC targets (Table 3).

**Table 2:** Summary results for the specificity panel.

Organism	IC (Ct)	LDT-Quant sgRNA	IC (Ct)	LDT-Quant VLCoV
**Adenovirus 3**	33.4	TND	33.5	TND
**Adenovirus 7a**	33.6	TND	33.8	TND
**Coronavirus 229E**	33.5	TND	33.5	TND
**CoronavirusNL63**	33.8	TND	33.7	TND
**Influenza A**	33.6	TND	33.5	TND
**Influenza B**	33.4	TND	33.4	TND
**Para Influenza 1**	34.1	TND	34.0	TND
**Para Influenza 2**	33.7	TND	33.9	TND
**Para Influenza 3**	33.7	TND	33.6	TND
***Bordetella pertussis***	33.7	TND	33.6	TND
***Candida albicans***	33.7	TND	33.6	TND
***Chlamydia pneumoniae***	33.5	TND	33.4	TND
**Human coronavirus OC43**	34.0	TND	33.8	TND
***Mycoplasmapneumoniae***	33.5	TND	33.9	TND
**Middle East respiratory syndrome Virus (NATMERS-ST MERS)**	33.5	TND	33.9	TND
**Severe acute respiratory syndrome Virus 1 (NATSARS-ST SARS1)**	33.4	TND	33.4	TND
***Pseudomonas aeruginosa***	34.0	TND	33.6	TND
**Rhinovirus**	33.3	TND	33.6	TND
**Respiratory Syncytial Virus-A**	33.4	TND	33.5	TND
**Respiratory Syncytial Virus -B**	33.5	TND	33.6	TND
***Staphylococcus epidermidis***	33.5	TND	33.5	TND
***Streptococcus pneumoniae***	33.3	TND	33.8	TND
***Streptococcus salivarius***	33.2	TND	33.7	TND
***Haemophilus influenzae***	33.2	TND	33.3	TND
***Pneumocystis carinii***	33.9	TND	33.3	TND
***Streptococcus pyogenes***	33.5	TND	33.3	TND
***Legionella pneumophila***	33.8	TND	33.7	TND
***Mycobacterium***. ***tuberculosis (DNA)***	34.1	TND	34.5	TND
***Human coronavirusHKU1 (RNA)***	33.5	TND	33.2	TND

IC: Internal Control; Ct: Cycle ThresholdTND: Target Not Detected

**Table 3:** Analytical interference results.

Organism	Viral Load Log_10_ (Copies/mL)	Difference From Baseline	IC (Ct)	Difference From Baseline
**Baseline 1 (Mean, N = 3)**	**4.84**	**N/A**	**33.96**	**N/A**
**Adenovirus 3**	4.70	-0.14	33.8	-0.16
**Adenovirus 7a**	4.88	0.04	33.7	-0.26
**Coronavirus NL63**	4.69	-0.15	33.7	-0.26
**Influenza A**	4.78	-0.06	33.5	-0.46
**Influenza B**	4.58	-0.26	33.5	-0.46
**Para Influenza 1**	4.78	-0.06	33.4	-0.56
**Para Influenza 2**	4.85	0.01	33.2	-0.76
**Human coronavirus OC43**	4.84	0.00	33.5	-0.46
***Mycoplasma pneumoniae***	4.65	-0.19	33.5	-0.46
**Severe acute respiratory syndrome Virus 1 (NATSARS-ST SARS1)**	4.81	-0.03	33.6	-0.36
***Pseudomonas aeruginosa***	4.59	-0.25	34.1	0.14
**Rhinovirus**	4.65	-0.19	33.8	-0.16
**Respiratory Syncytial Virus -A**	4.57	-0.27	33.9	-0.06
***Staphylococcus epidermidis***	4.81	-0.03	33.2	-0.76
***Streptococcus pneumoniae***	4.64	-0.20	33.8	-0.16
***Streptococcus salivarius***	4.75	-0.09	33.7	-0.26
***Haemophilus influenza***	4.68	-0.16	34.1	0.14
***Pneumocystis carinii***	4.66	-0.18	33.3	-0.66
***Streptococcus pyogenes***	4.87	0.03	34.0	0.04
***Mycobacteruim tuberculosis (DNA)***	4.65	-0.19	34.1	0.14
***Human coronavirus HKU1 (RNA)***	4.66	-0.18	33.9	-0.06
**Baseline 2 (Mean, N = 3)**	**4.71**	**NA**	**34.1**	**NA**
**Middle East respiratory syndrome Virus (NATMERS-ST MERS)**	4.73	-0.02	33.6	-0.5
**Baseline 3 (Mean, N = 3) **	**4.62**	**NA**	**33.83**	**NA**
***Bordetellapertussis (Mean***, ***N = 3)***	4.58	-0.04	33.63	-0.20
***Candida albicans (Mean***, ***N = 3) ***	4.66	0.04	33.47	-0.36
***Chlamydia pneumoniae (Mean***, ***N = 3) ***	4.68	0.06	33.67	-0.16
*** Human coronavirus 229E (Mean***, ***N = 3)***	4.67	0.05	33.60	-0.23
***Legionella pneumophila (Mean***, ***N = 3)***	4.58	-0.04	33.63	-0.20
***Para Influenza 3 (Mean***, ***N = 3)***	4.59	-0.03	33.83	0.00
**Respiratory Syncytial Virus *-B (Mean***, ***N = 3)***	4.66	0.04	34.00	0.17

NA: Not applicable, IC: Internal Control, Ct: Cycle Threshold

### Assay precision

**Fig 2:**
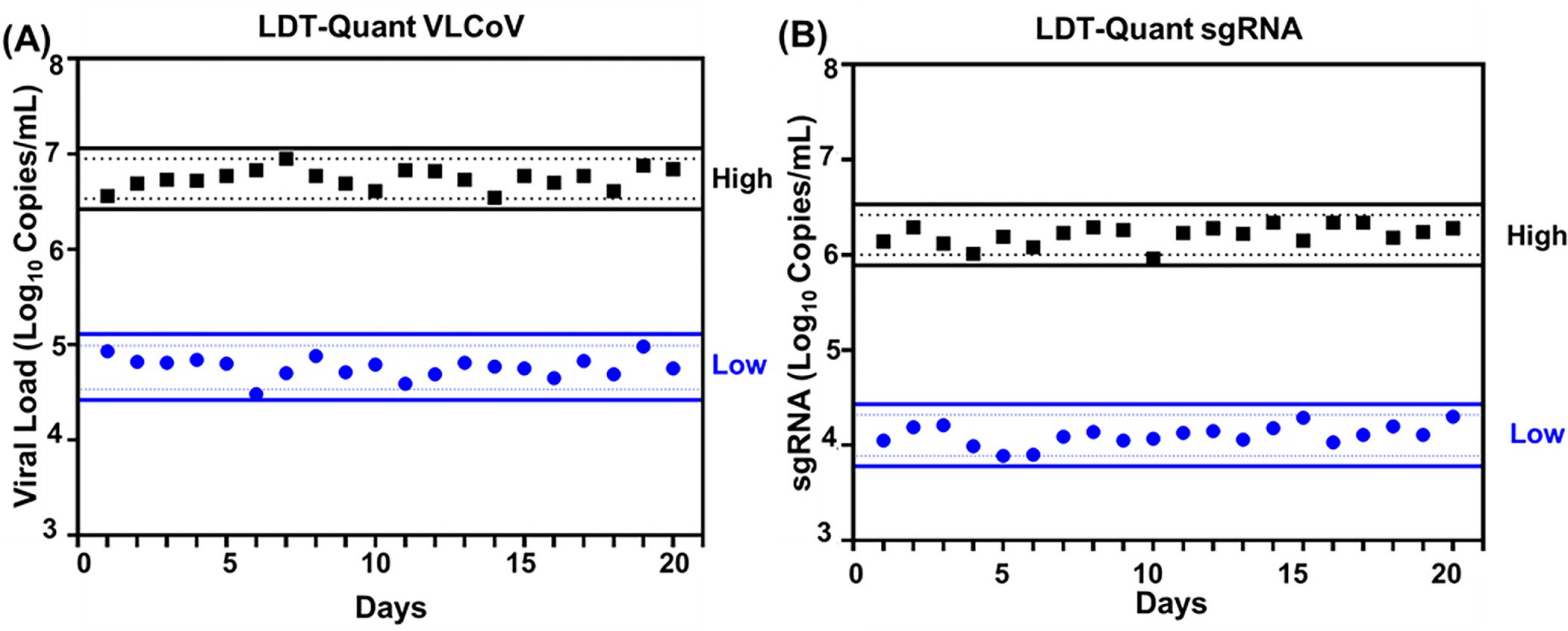
Precision of (A) LDT-Quant VLCoV and (B) LDT-Quant sgRNA assays positive extraction run controls. The precision of the High (in black) and Low (in blue) extraction controls is shown in a Levey-Jennings plot for 20 assay runs on log10-transformed values. Solid and dotted lines indicate ±3SD and ±2SD respectively.

Precision of the LDT-Quant VLCoV and LDT-Quant sgRNA assays was evaluated first based on results of the Low (1E4 Copies/mL) and High Extraction (1E6 Copies/mL) controls included for each assay. A Levey-Jennings analysis of 20 separate runs for each assay demonstrated the values for the High and Low run controls were consistent between runs: average of 6.74 (±0.33); 4.76 (±0.33) and 6.21 (±0.33); 4.11 (±0.33) Log_10_ Copies/mL for the LDT-Quant VLCoV and LDT-Quant sgRNA assays, respectively (Fig 2A and 2B). No amplification signal was detected for any negative run controls (N = 80) whose results were reported as “Target Not Detected” (TND).

**Fig 3:**
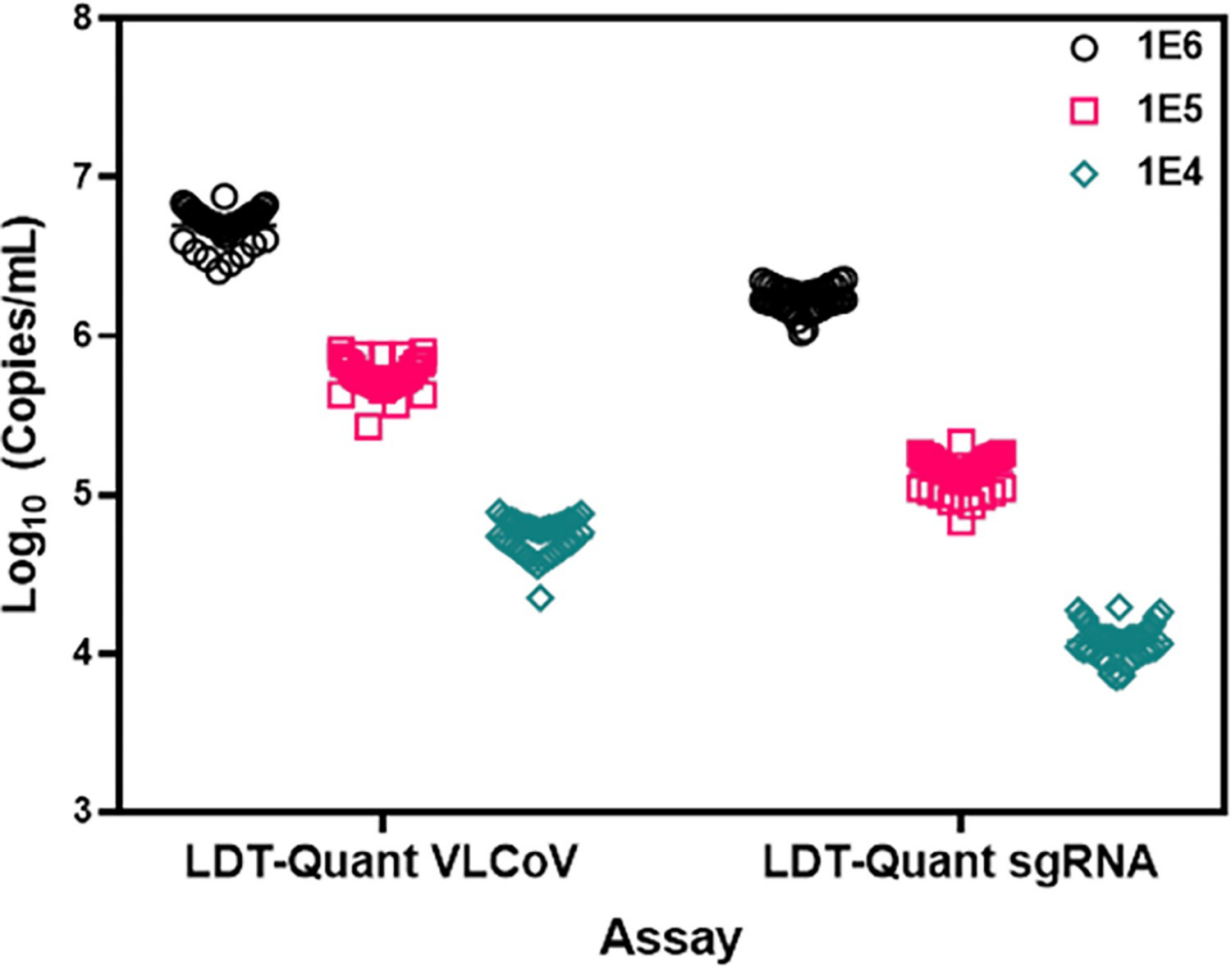
Viral load and sgRNA precision. Three concentrations of analyte spanning the reportable range were used to evaluate the precision of the LDT-Quant VLCoV and LDT-Quant sgRNA assays. Samples at each concentration (1E6, 1E5 and 1E4 Copies/mL) were tested in duplicate in one run per day over 20 days using two operators.

Precision for each assay was also evaluated by performing duplicate testing of three (3) panel members (1E6, 1E5 and 1E4), over 20 days (Fig 3). The CV for the LDT-Quant VLCoV and LDT-Quant sgRNA were acceptable, with the maximum CV of 2.5% and 2.6%, respectively (Tables 4 and 5).

**Table 4:** Precision results for the LDT-Quant VLCoV.

HI-SARS-CoV-2 Log_10_ (Copies/mL)	Viral Load Mean Log_10_ (Copies/mL)	Viral Load Within Run SD / CV	Viral Load Total SD / CV
**6.00**	6.69	0.138 / 2.1	0.138/ 2.1
**5.00**	5.74	0.115 / 2.0	0.136 / 2.4
**4.00**	4.75	0.118 / 2.5	0.118 / 2.5

**Table 5:** Precision results for the LDT-Quant sgRNA.

ARQ sgE Log_10_ (copies/mL)	sgRNA Mean Log_10_ (copies/mL)	sgRNA Within Run SD / CV	sgRNA Total SD / CV
**6.00**	6.23	0.094 / 1.5	0.094 / 1.5
**5.00**	5.14	0.093 / 1.8	0.106 / 2.1
**4.00**	4.08	0.091 / 2.2	0.108 / 2.6

### Linearity

**Fig 4:**
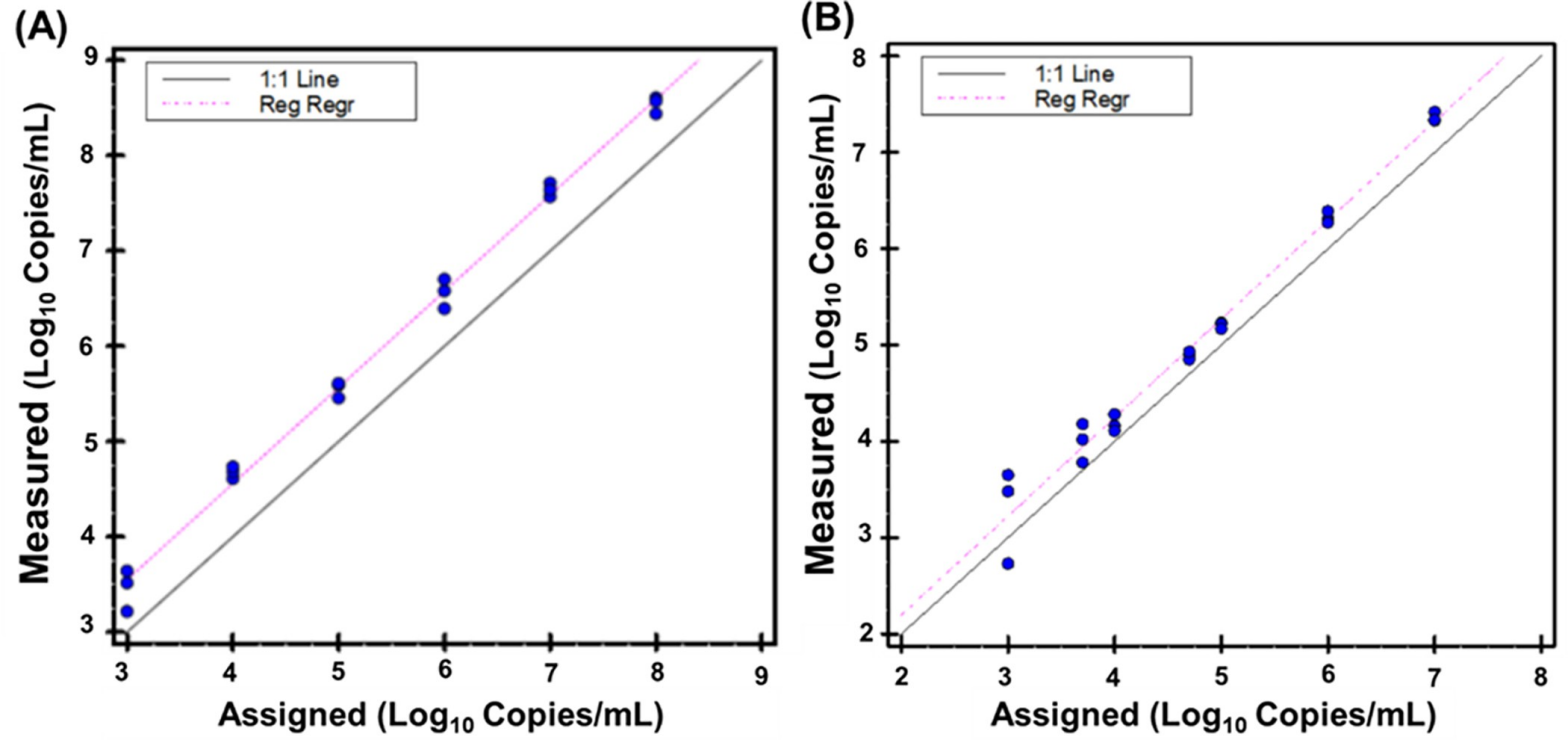
Plot of results from the (A) LDT-Quant VLCoV and (B) LDT-Quant sgRNA assays linearity experiment to determine reportable range. Assigned values (converted to Log_10_) were plotted on the x-axis versus measured values (converted to Log_10_) on the y-axis using EP Evaluator CLSI EP6 Linearity module based upon Log_10_ (Copies/mL).

Linearity of the LDT-Quant VLCoV assay was evaluated on serial dilutions of heat inactivated SARS-CoV-2 (ATCC). A panel consisting of six (6) concentrations serially diluted from 8 to 3 Log_10_ Copies/mL was prepared and tested in triplicate on a single run. The results demonstrated excellent linearity with a regression slope of 1.009 verifying the linearity throughout the range evaluated (Fig 4A). The linearity of the LDT-Quant sgRNA assay was evaluated using custom made sgRNA for the E gene. A panel consisting of seven (7) concentrations of ARQ sgE serially diluted from 7 to 3 Log_10_ Copies/mL was prepared and tested in triplicate on a single run. The results demonstrated acceptable linearity as well with a regression slope of 1.024 (Fig 4B).

### Lower Limit of Detection (LLOD)

**Fig 5:**
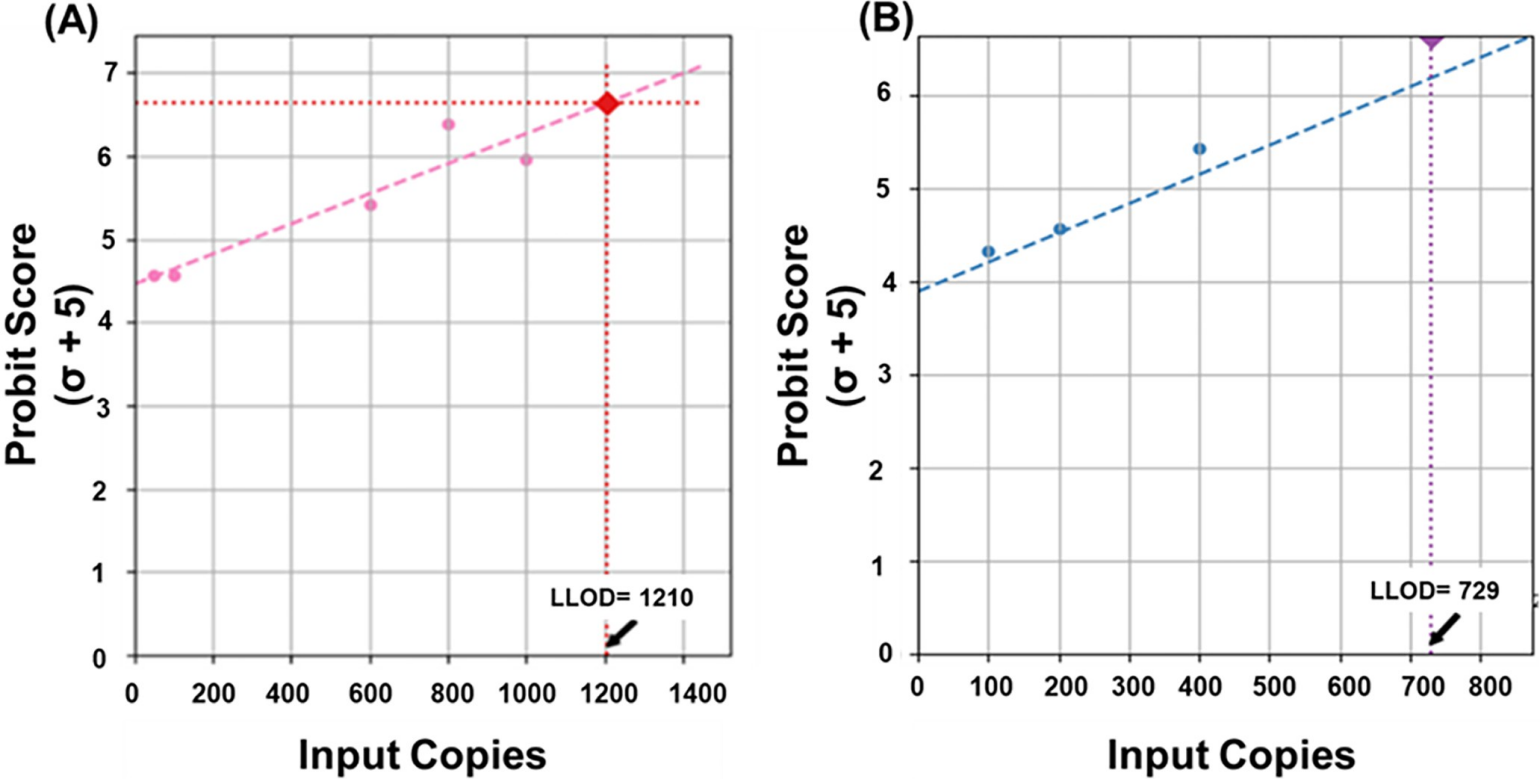
Probit Analysis for the (A) LDT-Quant VLCoV and (B) LDT-Quant sgRNA assays. The LLOD results were submitted to a Python script that performs a Linear Regression fit of the probit score in terms of the quantities for VL or sgRNA (in Copies/mL). The LLOD is extrapolated from the curve and as indicated by a black arrow.

The limit of detection for the LDT-Quant VLCoV and LDT-Quant sgRNA assays was evaluated independently for each assay by replicate measurements of HI-SARS-CoV-2 or ARQ sgE dilutions ranging from 500,000 to 10 Copies/mL (Table 6A and 6B). For the LDT-Quant VLCoV assay, all replicates (16/16) were detected at and above 2,000 Copies/mL HI-SARS-CoV-2 while only 13/16 replicates were detected at 1,000 Copies/mL HI-SARS-CoV-2 (Table 6A). None of the 14 replicates at 10 Copies/mL were detected. For the LDT-Quant sgRNA, 12 of the 12 replicates were detected at and above 800 Copies/mL ARQ sgE while only 8/12 replicates were detected at 400 Copies/mL (Table 6B). None of the twelve (12) replicates at 50 Copies/mL and none of the 12 replicates at 10 Copies/mL were detected. Analysis of results using the Probit Analysis (Python code developed in-house) indicated that the assays’ LLOD at 95% Confidence Interval (CI) are 1206 and 729 Copies/mL for the LDT-Quant VLCoV and LDT-Quant sgRNA assays, respectively (Fig 5).

**Table 6:** A and B: LLOD data for the LDT-Quant VLCoV (A) and LDT-Quant sgRNA (B) assays.

**(A)**		
**HI SARS-CoV-2 Copies/mL**	**Viral Load Detected Ratio**	**Viral Load % Detected**
**500,000**	16 / 16	100%
**100,000**	16 / 16	100%
**50,000**	16 / 16	100%
**10,000**	16 / 16	100%
**2,000**	16 / 16	100%
**1,000**	13 / 16	81.25%
**800**	15 / 16	93.75%
**600**	11 / 16	68.75%
**100**	5 / 16	31.25%
**50**	5 / 14	35.71%
**10**	0 / 14	0%
**(B)**		
**ARQ sgE Copies/mL**	**sgRNA Detected Ratio**	**sgRNA % Detected**
**10,000**	12/12	100%
**5,000**	12/12	100%
**1,000**	12/12	100%
**800**	12/12	100%
**400**	8/12	66.60%
**200**	4/12	33.33%
**100**	3/12	25%
**50**	0/12	0%
**10**	0/12	0%

### Comparison to other qRT-PCR assays

As there is no quantitative or gold standard SARS-CoV-2 assay available, the performance of the LDT-Quant VLCoV and LDT-Quant sgRNA assays was compared first to the EUA approved assay (Panther Fusion^®^ SARS-CoV-2 Assay by Hologic). Seventy SARS-CoV-2 positive samples and 100 negative samples, previously tested using the Panther Fusion^®^ EUA, were tested in our study. All positive specimens (70/70) were detected in the LDT-Quant VLCoV while only 64/70 positive samples were detected in LDT-Quant sgRNA. Positive Agreement (PPA), Negative Agreement (NPA) and Cohen’s Kappa were 100% for the LDT-Quant VLCoV (Table 7). For the LDT-Quant sgRNA, PPA, NPA and Cohen’s Kappa were 100%, 94.3%, and 92.6%, respectively.

**Table 7:** Positive Agreement (PPA), Negative Agreement (NPA) and Cohen’s Kappa.

	LDT-Quant VLCoV	LDT-Quant sgRNA
**Agreement**	100% (97.8 to 100%)	96.5% (92.5% to 98.4%)
**Positive Agreement**	100%	100%
**Negative Agreement**	100%	94.3%
**Cohen’s Kappa**	100%	92.6% (86.8 to 98.4%)

**Fig 6:**
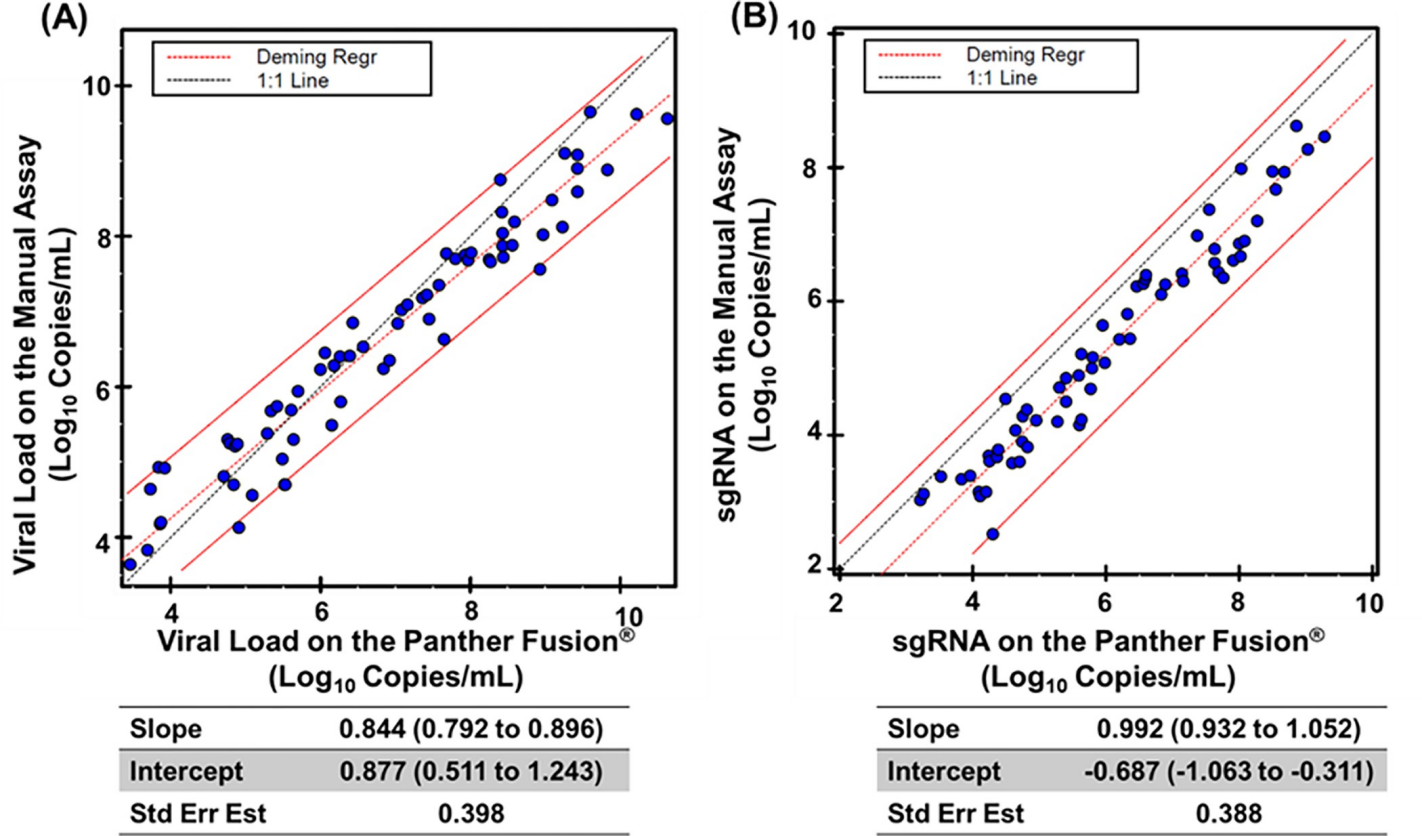
Quantitative Method Comparison for the (A) LDT-Quant VLCoV and (B) LDT-Quant sgRNA. Results of the Manual Assay (RUO only) were compared to the Panther Fusion^®^ LDT and plotted in EP Evaluator (Alternate Method comparison module). Deming regressions are shown below the graphs with 95% CI between parentheses.

Quantitative results were also evaluated against the results of the manual, research use only (RUO) version of the assay [23] with only samples quantified in both assays included in the analysis. The LDT-Quant VLCoV and LDT-Quant sgRNA assays correlate well with the viral load and sgRNA Manual Assays (slopes of 0.84 and 0.992 respectively) (Fig 6A and 6B). One noted difference between the manual assay and the Panther assay is the requirement of RNA dilution for the manual assay if the result is over log 8 while the Panther assay does not saturate until results are equal to / greater than log 9.

### Kinetics of viral load and sgRNA

**Fig 7:**
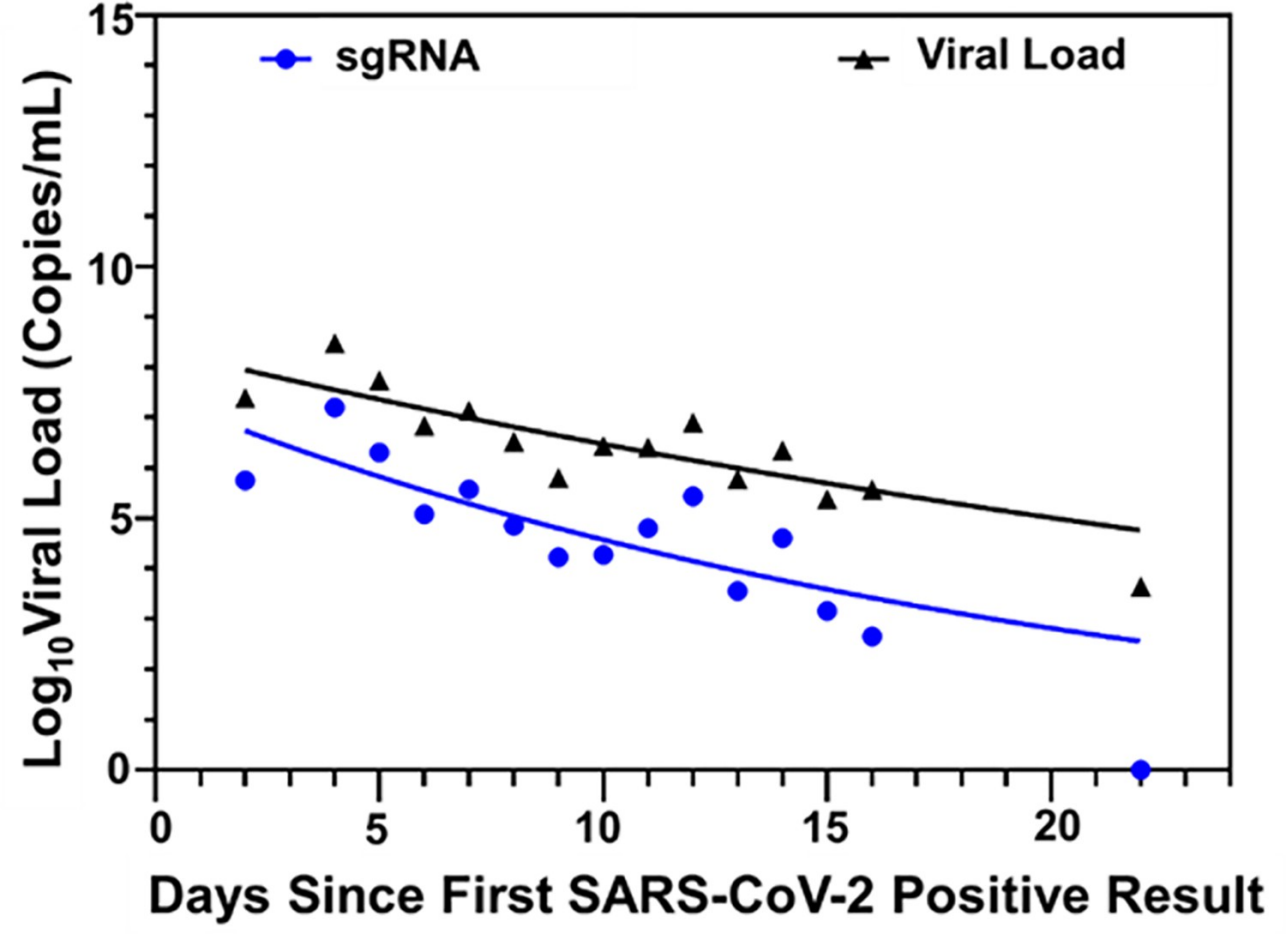
Viral trajectories of sgRNA and genomic viral loads. Viral Load and sgRNA were quantified on the Panther Fusion^®^ and average quantities per day (Log 10 Copies/mL) were plotted (black triangle for Viral Load and blue circle for sgRNA). The curves were generated in GraphPad with exponential growth model and indicate the dynamic tendency of viral load in analyzed samples.

To assess dynamics of the 70 SARS-CoV-2 positive samples over time, mean sgRNA and viral load results per day were calculated and plotted over the course of study period. Analysis in GraphPad of the exponential growth equation (nonlinear fit) demonstrated that sgRNA and VL had parallel kinetics and sgRNA could be detected 16 days post initial diagnostic test (Fig 7). In 45% of tested individuals (32/70), SARS-CoV-2 was detected post day 10 of initial diagnostic test for which 84% (27/32) had detectable sgRNA.

### Assay performance with SARS-CoV-2 Variants of Concern (VOC)

As the validation was performed using material derived from SARS-CoV-2 Washington strain (2019-nCoV/USA-WA1/2020) performance of the primers/probe sets was evaluated against VOCs; results demonstrated that amplification and quantification performance was not affected when using SARS-CoV-2 Alpha, Beta, Delta, and Omicron variants (Table 8).

**Table 8:** Performance of the LDT-Quant sgRNA and LDT-Quant VLCoV using variants of concern (VOC).

VOC	Vendor, Catalog ID and Lot Number	Expected	Quantified
		Log_10_ (Copies/mL) as quantified by Digital Droplet PCR	LDT-Quant VLCoV (Log_10_ Copies/mL)	LDT-Quant sgRNA (Log_10_ Copies/mL)
**SARS-CoV-2 Alpha**	ATCC, VR-3326HK™, 70043248	5.78	6.61	4.73
**SARS-CoV-2 Beta**	ATCC, VR-3327HK™, 70045506	6.2	6.85	4.67
**SARS-CoV-2 Delta**	ATCC, VR-3342HK™, 70048932	6.18	6.84	5.07
**SARS-CoV-2 Omicron**	ATCC, VR-3347HK™, 70050282	6.28	6.72	5.29

## Discussion

The COVID-19 pandemic has rapidly accelerated the development and implementation of new molecular approaches for diagnosis of SARS-CoV-2 by increasing test sensitivity and decreasing test turn-around time. These advances highlight the need for quantitative results to better understand the temporal evolution of viral load, duration of infectivity, the likelihood of community transmission, and potential for predicting disease severity [25, 26].

The ever-expanding number of SARS-CoV-2 tests with different performance characteristics and limitations [25] in conjunction with the need to minimize duration of isolation within the military settings drove the development of a high throughput, Panther Fusion® (Hologic) SARS-CoV-2 sugbenomic RNA (sgRNA) assay for detection and quantitation of sgRNA.

In this study, two quantitative RT-PCR assays were developed and validated to measure SARS-CoV-2 sgRNA and genomic viral loads (E target). The limits of detection were 1206 and 729 Copies/mL for the LDT-Quant VLCoV and LDT-Quant sgRNA assays, respectively. Analytical specificity studies demonstrated neither cross-reactivity nor interference with common respiratory pathogens.

Assays were assessed using a small cohort of clinical samples totaling 170 accuracy samples (70 samples positive for SARS-CoV-2 from asymptomatic individuals and 100 samples negative for SARS-CoV-2), previously tested by Hologic Panther Fusion^®^ EUA. Results showed acceptable accuracy:100% for the LDT-Quant VLCoV and 92.6% for the LDT-Quant sgRNA. The slightly lower accuracy for the LDT-Quant sgRNA is likely due to the lower abundance or absence of sgRNA in low viral load samples and /or due to the lower volume of input material for the Panther Fusion® as compared to the RUO Manual Assay. Alpha, Beta, Delta and Omicron SARS-CoV-2 VOCs were accurately quantified for both targets attesting to the assay’s robustness in detection of evolving VOCs. This finding contradicts earlier report that E gene variant alters the analytical sensitivity characteristics of viral detection [27] and is explained by our target design (Nucleotide 26,372 is not in the sequence of the primers/probe set for TAL E3). Moreover, computational modeling showed that the E gene is less prone to genetic variation compared to other SARS-CoV-2 genes [28].

As reference materials were not commercially available for E sgRNA and the variability of sgRNA in commercial Heat Inactivated SARS-CoV-2 reagents lots from commercial sources were major hurdles in this study, an armored RNA harboring the full sequence of sgRNA for the E gene was custom manufactured by Asuragen. This Armored RNA was effectively leveraged for the creation of assay controls, linearity studies, and development of LLOD panels. Although designing the armored RNA resulted in a delay in analytical validation, overall it provides reliable assay controls and enhances the ability to evaluate sgRNA dynamics in future studies.

Limitations of this study include the small sample size, e.g., 70 SARS-CoV-2 samples from a serially tested cohort totaling over 1100 specimens representing 229 recruits isolated for COVID-19 [23]. While samples spanned low, moderate, and high viral load ranges, results could not be correlated to infectivity by viral culture since all samples were archival frozen samples.

Our study focused on analytical performance of the two (2) novel LDTs, it did not validate sgRNA as a biomarker for infectiousness. Albeit a manual version of the LDT-Quant sgRNA assay leveraged for serial testing in support of an enhanced surveillance public health project demonstrated that a cycle threshold below 30.49 on the Panther Fusion^®^ assay or manual sgRNA assay result above 3.09 Log_10_ Copies/mL demonstrated highest sensitivity and specificity for identification of active infection in military recruits. Additionally, use of this same assay in support of SARS-CoV-2 vaccine efficacy trials in Non-human Primates [29–31] and Hamsters [32] provided evidence that sgRNA is a useful biomarker for virus replication/clearance in these animal models. These results suggest that further studies are required to evaluate potential use of these assays as biomarkers for therapeutic management and/or as surrogate markers to predict the duration of infectious viral shedding; thus, reducing the isolation time in both public and military settings. Clinical utility of the LDT-Quant sgRNA is a critical priority in our laboratory; studies leveraging sequential samples from a larger longitudinal cohort study are planned.

## Supporting information

S1 Table RNA thermal profile used for the LDT-Quant VLCoV and LDT-Quant sgRNA assays. (DOCX)

S2 Table PPRs (Primers Probes Reagents) for the LDT-Quant VLCoV and LDT-Quant sgRNA assays. (DOCX)

S3 Table List of organisms used to create specificity and sensitivity panels. (DOCX)

S4 Table Summary of standard curves parameters. (DOCX)

S1 Data (XLSX)

## References

[1] Green DA, Zucker J, Westblade LF, Whittier S, Rennert H, Velu P, et al. Clinical Performance of SARS-CoV-2 Molecular Tests. J Clin Microbiol. 2020;58(8). Epub 2020/06/10. doi: 10.1128/JCM.00995-20 ; PubMed Central PMCID: PMC7383556. 32513858 PMC7383556

[2] Sah P, Fitzpatrick MC, Zimmer CF, Abdollahi E, Juden-Kelly L, Moghadas SM, et al. Asymptomatic SARS-CoV-2 infection: A systematic review and meta-analysis. Proc Natl Acad Sci U S A. 2021;118(34). Epub 2021/08/12. doi: 10.1073/pnas.2109229118 ; PubMed Central PMCID: PMC8403749. 34376550 PMC8403749

[3] Lefeuvre C, Pivert A, Przyrowski E, Bouthry E, Darviot E, Mahieu R, et al. Comparison of performance between three SARS-CoV-2 molecular assays (Aptima, Laboratory Developed Test-Fusion, and R-GENE(R)) with special attention to turnaround time, a key point in laboratory management. J Med Virol. 2022. Epub 2022/02/26. doi: 10.1002/jmv.27675 . 35211992 PMC9088664

[4] Jang S, Rhee JY, Wi YM, Jung BK. Viral kinetics of SARS-CoV-2 over the preclinical, clinical, and postclinical period. Int J Infect Dis. 2021;102:561–5. Epub 2020/11/08. doi: 10.1016/j.ijid.2020.10.099 ; PubMed Central PMCID: PMC7642732. 33160066 PMC7642732

[5] Arons MM, Hatfield KM, Reddy SC, Kimball A, James A, Jacobs JR, et al. Presymptomatic SARS-CoV-2 Infections and Transmission in a Skilled Nursing Facility. N Engl J Med. 2020;382(22):2081–90. Epub 2020/04/25. doi: 10.1056/NEJMoa2008457 ; PubMed Central PMCID: PMC7200056. 32329971 PMC7200056

[6] Bullard J, Dust K, Funk D, Strong JE, Alexander D, Garnett L, et al. Predicting Infectious Severe Acute Respiratory Syndrome Coronavirus 2 From Diagnostic Samples. Clin Infect Dis. 2020;71(10):2663–6. Epub 2020/05/23. doi: 10.1093/cid/ciaa638 ; PubMed Central PMCID: PMC7314198. 32442256 PMC7314198

[7] Lu J, Peng J, Xiong Q, Liu Z, Lin H, Tan X, et al. Clinical, immunological and virological characterization of COVID-19 patients that test re-positive for SARS-CoV-2 by RT-PCR. EBioMedicine. 2020;59:102960. Epub 2020/08/28. doi: 10.1016/j.ebiom.2020.102960 ; PubMed Central PMCID: PMC7444471. 32853988 PMC7444471

[8] Davies M, Bramwell LR, Jeffery N, Bunce B, Lee BP, Knight B, et al. Persistence of clinically relevant levels of SARS-CoV2 envelope gene subgenomic RNAs in non-immunocompromised individuals. Int J Infect Dis. 2022;116:418–25. Epub 2021/12/11. doi: 10.1016/j.ijid.2021.12.312 ; PubMed Central PMCID: PMC8757659. 34890790 PMC8757659

[9] Wolfel R, Corman VM, Guggemos W, Seilmaier M, Zange S, Muller MA, et al. Virological assessment of hospitalized patients with COVID-2019. Nature. 2020;581(7809):465–9. Epub 2020/04/03. doi: 10.1038/s41586-020-2196-x . 32235945

[10] Wu B, White KA. Uncoupling RNA virus replication from transcription via the polymerase: functional and evolutionary insights. EMBO J. 2007;26(24):5120–30. Epub 2007/11/24. doi: 10.1038/sj.emboj.7601931 ; PubMed Central PMCID: PMC2140117. 18034156 PMC2140117

[11] Kim D, Lee JY, Yang JS, Kim JW, Kim VN, Chang H. The Architecture of SARS-CoV-2 Transcriptome. Cell. 2020;181(4):914–21 e10. Epub 2020/04/25. doi: 10.1016/j.cell.2020.04.011 ; PubMed Central PMCID: PMC7179501. 32330414 PMC7179501

[12] Wu F, Zhao S, Yu B, Chen YM, Wang W, Song ZG, et al. A new coronavirus associated with human respiratory disease in China. Nature. 2020;579(7798):265–9. Epub 2020/02/06. doi: 10.1038/s41586-020-2008-3 ; PubMed Central PMCID: PMC7094943. 32015508 PMC7094943

[13] van Kampen JJA, van de Vijver D, Fraaij PLA, Haagmans BL, Lamers MM, Okba N, et al. Duration and key determinants of infectious virus shedding in hospitalized patients with coronavirus disease-2019 (COVID-19). Nat Commun. 2021;12(1):267. Epub 2021/01/13. doi: 10.1038/s41467-020-20568-4 ; PubMed Central PMCID: PMC7801729. 33431879 PMC7801729

[14] Saharia KK, Ramelli SC, Stein SR, Roder AE, Kreitman A, Banakis S, et al. Successful lung transplantation using an allograft from a COVID-19-recovered donor: a potential role for subgenomic RNA to guide organ utilization. Am J Transplant. 2023;23(1):101–7. Epub 2023/01/26. doi: 10.1016/j.ajt.2022.09.001 ; PubMed Central PMCID: PMC9833374. 36695611 PMC9833374

[15] Alonso-Navarro R, Cuesta G, Santos M, Cardozo C, Rico V, Garcia-Pouton N, et al. Qualitative Subgenomic RNA to Monitor the Response to Remdesivir in Hospitalized Patients With Coronavirus Disease 2019: Impact on the Length of Hospital Stay and Mortality. Clin Infect Dis. 2023;76(1):32–8. Epub 2022/09/14. doi: 10.1093/cid/ciac760 ; PubMed Central PMCID: PMC9494412. 36097825 PMC9494412

[16] Administration USFaD. Panther Fusion SARS-CoV-2 Emergency Use Authorization. 2020.

[17] Kulis-Horn RK, Tiemann C. Evaluation of a laboratory-developed test for simultaneous detection of norovirus and rotavirus by real-time RT-PCR on the Panther Fusion(R) system. Eur J Clin Microbiol Infect Dis. 2020;39(1):103–12. Epub 2019/09/12. doi: 10.1007/s10096-019-03697-7 ; PubMed Central PMCID: PMC6962121. 31506730 PMC6962121

[18] Stellrecht KA, Cimino JL, Maceira VP. The Panther Fusion System with Open Access Functionality for Laboratory-Developed Tests for Influenza A Virus Subtyping. J Clin Microbiol. 2020;58(6). Epub 2020/04/02. doi: 10.1128/JCM.00188-20 ; PubMed Central PMCID: PMC7269382. 32229600 PMC7269382

[19] Voermans JJC, Mulders D, Pas SD, Koopmans MPG, van der Eijk AA, Molenkamp R. Performance evaluation of the Panther Fusion(R) respiratory tract panel. J Clin Virol. 2020;123:104232. Epub 2019/12/24. doi: 10.1016/j.jcv.2019.104232 ; PubMed Central PMCID: PMC7172494. 31869661 PMC7172494

[20] Cordes AK, Heim A. Rapid random access detection of the novel SARS-coronavirus-2 (SARS-CoV-2, previously 2019-nCoV) using an open access protocol for the Panther Fusion. J Clin Virol. 2020;125:104305. Epub 2020/03/07. doi: 10.1016/j.jcv.2020.104305 ; PubMed Central PMCID: PMC7129486. 32143123 PMC7129486

[21] Migueres M, Mengelle C, Dimeglio C, Didier A, Alvarez M, Delobel P, et al. Saliva sampling for diagnosing SARS-CoV-2 infections in symptomatic patients and asymptomatic carriers. J Clin Virol. 2020;130:104580. Epub 2020/08/12. doi: 10.1016/j.jcv.2020.104580 ; PubMed Central PMCID: PMC7405829. 32781366 PMC7405829

[22] Dust K, Hedley A, Nichol K, Stein D, Adam H, Karlowsky JA, et al. Comparison of commercial assays and laboratory developed tests for detection of SARS-CoV-2. J Virol Methods. 2020;285:113970. Epub 2020/09/14. doi: 10.1016/j.jviromet.2020.113970 ; PubMed Central PMCID: PMC7482591. 32920028 PMC7482591

[23] Hakre S, Elakhal Naouar I, King DB, Burns JL, Jackson KN, Krauss SW, et al. Virological and serological assessment of U.S. Army trainees isolated for COVID-19. J Infect Dis. 2022. Epub 2022/05/12. doi: 10.1093/infdis/jiac198 . 35543272 PMC9129211

[24] Hakre S, Sanborn AD, Krauss SW, Burns JL, Jackson KN, McCauley MD, et al. Serological and RT-PCR Surveillance for COVID-19 in an Asymptomatic US Army Trainee Population. Open Forum Infect Dis. 2021;8(9):ofab407. Epub 2021/09/14. doi: 10.1093/ofid/ofab407 ; PubMed Central PMCID: PMC8418190. 34514020 PMC8418190

[25] Cherkaoui D, Heaney J, Huang D, Byott M, Miller BS, Nastouli E, et al. Clinical Validation of a Rapid Variant-Proof RT-RPA Assay for the Detection of SARS-CoV-2. Diagnostics (Basel). 2022;12(5). Epub 2022/05/29. doi: 10.3390/diagnostics12051263 . 35626420 PMC9141210

[26] Jayakody H, Kiddle G, Perera S, Tisi L, Leese HS. Molecular diagnostics in the era of COVID-19. Anal Methods. 2021;13(34):3744–63. Epub 2021/09/03. doi: 10.1039/d1ay00947h . 34473144

[27] Tahan S, Parikh BA, Droit L, Wallace MA, Burnham CD, Wang D. SARS-CoV-2 E Gene Variant Alters Analytical Sensitivity Characteristics of Viral Detection Using a Commercial Reverse Transcription-PCR Assay. J Clin Microbiol. 2021;59(7):e0007521. Epub 2021/04/28. doi: 10.1128/JCM.00075-21 ; PubMed Central PMCID: PMC8218754. 33903167 PMC8218754

[28] Ashoor D, Ben Khalaf N, Marzouq M, Jarjanazi H, Chlif S, Fathallah MD. A Computational Approach to Evaluate the Combined Effect of SARS-CoV-2 RBD Mutations and ACE2 Receptor Genetic Variants on Infectivity: The COVID-19 Host-Pathogen Nexus. Front Cell Infect Microbiol. 2021;11:707194. Epub 2021/08/27. doi: 10.3389/fcimb.2021.707194 ; PubMed Central PMCID: PMC8381355. 34434902 PMC8381355

[29] Joyce MG, King HAD, Naouar IE, Ahmed A, Peachman KK, Cincotta CM, et al. Efficacy of a Broadly Neutralizing SARS-CoV-2 Ferritin Nanoparticle Vaccine in Nonhuman Primates. bioRxiv. 2021. Epub 2021/04/02. doi: 10.1101/2021.03.24.436523 ; PubMed Central PMCID: PMC8010721. 33791694 PMC8010721

[30] King HAD, Joyce MG, Lakhal-Naouar I, Ahmed A, Cincotta CM, Subra C, et al. Efficacy and breadth of adjuvanted SARS-CoV-2 receptor-binding domain nanoparticle vaccine in macaques. Proc Natl Acad Sci U S A. 2021;118(38). Epub 2021/09/03. doi: 10.1073/pnas.2106433118 ; PubMed Central PMCID: PMC8463842. 34470866 PMC8463842

[31] Johnston SC, Ricks KM, Lakhal-Naouar I, Jay A, Subra C, Raymond JL, et al. A SARS-CoV-2 Spike Ferritin Nanoparticle Vaccine Is Protective and Promotes a Strong Immunological Response in the Cynomolgus Macaque Coronavirus Disease 2019 (COVID-19) Model. Vaccines (Basel). 2022;10(5). Epub 2022/05/29. doi: 10.3390/vaccines10050717 . 35632473 PMC9145473

[32] Wuertz KM, Barkei EK, Chen WH, Martinez EJ, Lakhal-Naouar I, Jagodzinski LL, et al. A SARS-CoV-2 spike ferritin nanoparticle vaccine protects hamsters against Alpha and Beta virus variant challenge. NPJ Vaccines. 2021;6(1):129. Epub 2021/10/30. doi: 10.1038/s41541-021-00392-7 ; PubMed Central PMCID: PMC8553838. 34711815 PMC8553838

