# Supplementary material for: Analytical validation of quantitative SARS-CoV-2 subgenomic and viral load laboratory developed tests conducted on the Panther Fusion® (Hologic) with preliminary application to clinical samples
Source: PLoS One. 2023 Jun 29;18(6):e0287576. doi: 10.1371/journal.pone.0287576 (PMC10309597; doi:10.1371/journal.pone.0287576)
Supplement: S1 Table — (DOCX) [file pone.0287576.s001.docx]

**Analytical Validation of Quantitative SARS-CoV-2 Subgenomic and Viral Load Laboratory Developed Tests Conducted on the Panther Fusion® (Hologic) with Preliminary Application to Clinical Samples**

Ines Lakhal-Naouar, Holly R. Hack, Edgar Moradel, Amie Jarra, Hannah L. Grove, Rani M. Ismael, Steven Padilla, Dante Coleman, Jason Ouellette, Janice Darden, Casey Storme, Kristina K. Peachman, Tara L. Hall, Mark E. Huhtanen, Paul T. Scott, Shilpa Hakre, Linda L. Jagodzinski and Sheila A. Peel

**SUPPORTING INFORMATION**

**Table S1: RNA Thermal profile used for the LDT-Quant VLCoV and LDT-Quant sgRNA assays**

| Stage Name | 1: Holding Stage | | 2: Cycling Stage | |
| --- | --- | --- | --- | --- |
| Number Of Cycles | 1 | | 45 | |
| Step Name | Step 1 | Step 2 | Step 1 | Step 2 |
| Temperature (^O^C) | 46 | 95 | 95 | 60 |
| Duration (mm:ss) | 08:00 | 02:00 | 00:05 | 00:22 |
| Optics On |  |  |  | **On** |

mm: minutes, ss: seconds, ^O^C: degree Celsius
