# Supplementary material for: Analytical validation of quantitative SARS-CoV-2 subgenomic and viral load laboratory developed tests conducted on the Panther Fusion® (Hologic) with preliminary application to clinical samples
Source: PLoS One. 2023 Jun 29;18(6):e0287576. doi: 10.1371/journal.pone.0287576 (PMC10309597; doi:10.1371/journal.pone.0287576)
Supplement: S3 Table — (DOCX) [file pone.0287576.s003.docx]

**Analytical Validation of Quantitative SARS-CoV-2 Subgenomic and Viral Load Laboratory Developed Tests Conducted on the Panther Fusion® (Hologic) with Preliminary Application to Clinical Samples**

Ines Lakhal-Naouar, Holly R. Hack, Edgar Moradel, Amie Jarra, Hannah L. Grove, Rani M. Ismael, Steven Padilla, Dante Coleman, Jason Ouellette, Janice Darden, Casey Storme, Kristina K. Peachman, Tara L. Hall, Mark E. Huhtanen, Paul T. Scott, Shilpa Hakre, Linda L. Jagodzinski and Sheila A. Peel

**SUPPORTING INFORMATION**

**Table S3: List of organisms used to create specificity and sensitivity panels**

| Nomenclature | Organism | Cat No. | Source |
| --- | --- | --- | --- |
| *Adenovirus 3* | *Adenovirus 3* | 0810062CF | Zeptometrix |
| *Adenovirus 7a* | *Adenovirus 7a* | 0810021CF | Zeptometrix |
| *CoV229E* | Human coronavirus 229E | 0810229CF | Zeptometrix |
| *CoVNL63* | Human coronavirus HCOV-NL63 | 0810228CF | Zeptometrix |
| *Influenza A* | *Influenza A* | 0810506CF | Zeptometrix |
| *Influenza B* | *Influenza B* | 0810037CF | Zeptometrix |
| *Paraflu 1* | *Para Influenza 1* | 0810014CF | Zeptometrix |
| *Paraflu 2* | *Para Influenza 2* | 0810015CF | Zeptometrix |
| *Paraflu 3* | *Para Influenza 3* | 0810016CF | Zeptometrix |
| *B. pertussis* | *Bordetella pertussis* | 9797^TM^ | ATCC |
| *C. albicans* | *Candida Albicans* | 64124 | ATCC |
| *C. pneumoniae* | *Chlamydia pneumoniae* | VR-2282 | ATCC |
| *CoVOC43* | Human coronavirus OC43 | 0810024CF | Zeptometrix |
| *M. pneumoniae* | *Mycoplasma pneumoniae* | 15531-TTR | ATCC |
| *NATMERS-ST MERS* | MERS-CoV | NATMERS-ST | Zeptometrix |
| *NATSARS-ST SARS1* | Coronavirus SARS | NATSARS-ST | Zeptometrix |
| *P. aeruginosa* | *Pseudomonas aeruginosa* | 10145 | ATCC |
| *Human Rhinovirus* | *Human Rhinovirus* | VR-283 | ATCC |
| *RSV-A* | Respiratory syncytial virus-A | 0810040ACF | Zeptometrix |
| *RSV-B* | Respiratory syncytial virus-B | 0810450CF | Zeptometrix |
| *S. epidermidis* | *Staphylococcus epidermidis* | 14990 | ATCC |
| *S. pneumoniae* | *Streptococcus pneumoniae* | 49619 | ATCC |
| *S. salivarius* | *Streptococcus salivarius* | 25975 | ATCC |
| *H. influenza* | *Haemophilus influenzae* | 51907 | ATCC |
| *P. carinii* | *Pneumocystis carinii* | PRA-159 | ATCC |
| *S. pyogenes* | *Streptococcus pyogenes* | 12344 | ATCC |
| *L. pneumophila* | *Legionella pneumophila* | 33152 | ATCC |
| *M. tuberculosis (DNA)* | *Mycobacterium tuberculosis* | 25177DQ | ATCC |
| *HKU1 (RNA)* | Human Coronavirus HKU1 | VR-3262SD | ATCC |
